# Awake pancreaticoduodenectomy without intubation: exploring short-term clinicopathological outcomes of epidural vs. general anesthesia

**DOI:** 10.3389/fsurg.2025.1675019

**Published:** 2025-10-08

**Authors:** Iyad Hassan, Lina Hassan, Ibrahim Gamal, Mohamad Ibrahim, Wiam Hassan

**Affiliations:** 1 Department of Surgery, Burjeel Hospital, Abu Dhabi, United Arab Emirates; 2 Department of Anesthesia, Burjeel Hospital, Abu Dhabi, United Arab Emirates

**Keywords:** pancreatic cancer, pyloric preserved pancreaticoduodenectomy, general anesthesia, epidural anesthesia, oncological outcomes, surgical complications

## Abstract

**Background:**

There is some evidence to suggest that general anesthesia may influence oncological outcomes, such as survival and disease-free recurrence, in addition to surgical outcomes. This study compares the clinico-oncological outcomes of pancreatic cancer patients who had a pyloric-preserving pancreaticoduodenectomy (PPPD) under epidural anesthesia without endotracheal intubation (EA) and those who received general anesthesia (GA).

**Methods:**

A retrospective cohort investigation comparing pancreatic cancer patients with PPPD under GA and EA. The procedure's feasibility and 30-day clinico-pathological outcomes were evaluated between groups.

**Results:**

The ratio of males to females was 16:5. The mean age was 51 years (range 27–74 years). The median hospital stay was 12 days (range 7–60). In the GA group, thirteen patients had PPPD and one patient received total pancreatectomy with splenectomy (TPS). On the other hand, in the EA group, six patients received PPPD and two patients underwent TPS. The two groups had similar preoperative demographics, including ASA classification. Seven EA patients underwent successful surgery without GA conversion. Due to respiratory acidosis, one TPS patient was converted to GA before abdominal closure. Neither group had mortality or major cardio-pulmonary issues, with the exception of one case in the GA group who acquired COVID-19 while hospitalized and was ventilated for 10 days until completely recovering. Surgical complications occurred as follows: Two GA patients had pancreatic fistula type B, and one EA patient had a biliary leak, both treated conservatively. One GA patient needed a revision laparoscopy after an iatrogenic bowl perforation during IR drain insertion for chylous ascites on postoperative day 30. All cases had an R0 resection. The histological tumor stage was similar in both groups. The EA group had significantly more harvested lymph nodes and a higher number of lymph node metastases (*p* = 0.022 and *P* = 0.005, respectively).

**Conclusions:**

Pancreaticoduodenectomy with just epidural anesthesia and without endotracheal intubation can be performed safely in selected cases. It may decrease surgical complications without affecting oncological outcomes. Additional research is necessary to comprehend its actual advantages.

## 1 Introduction

Pancreatic cancer remains one of the most aggressive malignancies, with surgical resection serving as the only potentially curative treatment despite dismal long-term survival rates (1). Pancreaticoduodenectomy (PD) is central to curative therapy; however, the perioperative period is critical, as the anesthetic technique employed can significantly influence immune function and tumor biology. General anesthesia (GA) with endotracheal intubation triggers a robust neuroendocrine stress response, resulting in elevated catecholamines and pro-inflammatory cytokines that may impair natural killer cell function and promote tumor dissemination (2, 3). Moreover, GA induces significant alterations in the plasma metabolome, which can further impact tumor behavior (4). In contrast, epidural anesthesia (EA) attenuates these adverse responses, providing superior postoperative pain control and fostering a more favorable immunologic and metabolic milieu (5–7).

Emerging evidence suggests that the anesthetic technique used during oncologic surgery may significantly influence both perioperative and long-term cancer outcomes. GA with endotracheal intubation provokes a pronounced stress response—and is associated with deleterious metabolomic shifts—that may impair immune function and promote tumor dissemination, while EA mitigates this neuroendocrine response and may favorably modulate tumor biology (8–11).

Additionally, Chen et al. reported that intraoperative epidural Ropivacaine infusion positively impacted oncological outcomes in pancreatic cancer patients (12). However, a meta-analysis by Ang et al. neither supports nor refutes the association between the use of regional anesthesia and a lower incidence of cancer recurrence compared to GA in oncological resections (13).

Hence, optimal surgical stress management necessitates the implementation of the most effective anesthetic techniques. Potent pain management, prompt mobilization, and swift recovery are recommended to decrease the occurrence of complications as well as tumor recurrence. Historical studies by Nakashima et al. (14) and Ueo et al. demonstrated the feasibility of performing major abdominal surgery under EA without endotracheal intubation—even in elderly patients (15). Subsequent recent research, such as the pilot study on neuraxial anesthesia in hepato-pancreato-biliary surgery have provided additional evidence of the feasibility of the utilization of EA alone for complex hepatobiliary and pancreatic surgery (16). This prompted several research groups to assess the impact of epidural analgesia on short-term postoperative clinical and oncological outcomes in prospective controlled trials involving pancreatic surgeries (17, 18).

In light of these observations, this study aims to assess the feasibility and compare the early clinico-oncological outcomes in pancreatic cancer patients undergoing pylorus-preserving pancreaticoduodenectomy (PPPD) performed under epidural anesthesia without endotracheal intubation (EA) vs. those receiving general anesthesia (GA).

## 2 Materials and methods

### 2.1 Study design and patient selection

A retrospective cohort study was conducted at Burjeel Hospital, Abu Dhabi. The institutional review board approved this study, and informed consent was obtained from all patients. We included pancreatic cancer patients who underwent either PPPD or total pancreatectomy with or without splenectomy (TPS) between January 2015 and December 2022. Patient selection for epidural anesthesia (EA) vs. general anesthesia (GA) was based on a comprehensive preoperative assessment, which included anesthesiologist evaluation, patient preference, and the availability of M.I., an expert in regional anesthesia, who provided guidance on the feasibility of EA for each case. All patients who met the inclusion criteria and underwent pancreaticoduodenectomy at our institution during the study period were consecutively included

Patients were stratified as follows:

#### 2.1.1 Epidural anesthesia without Ga

**Figure 1:**
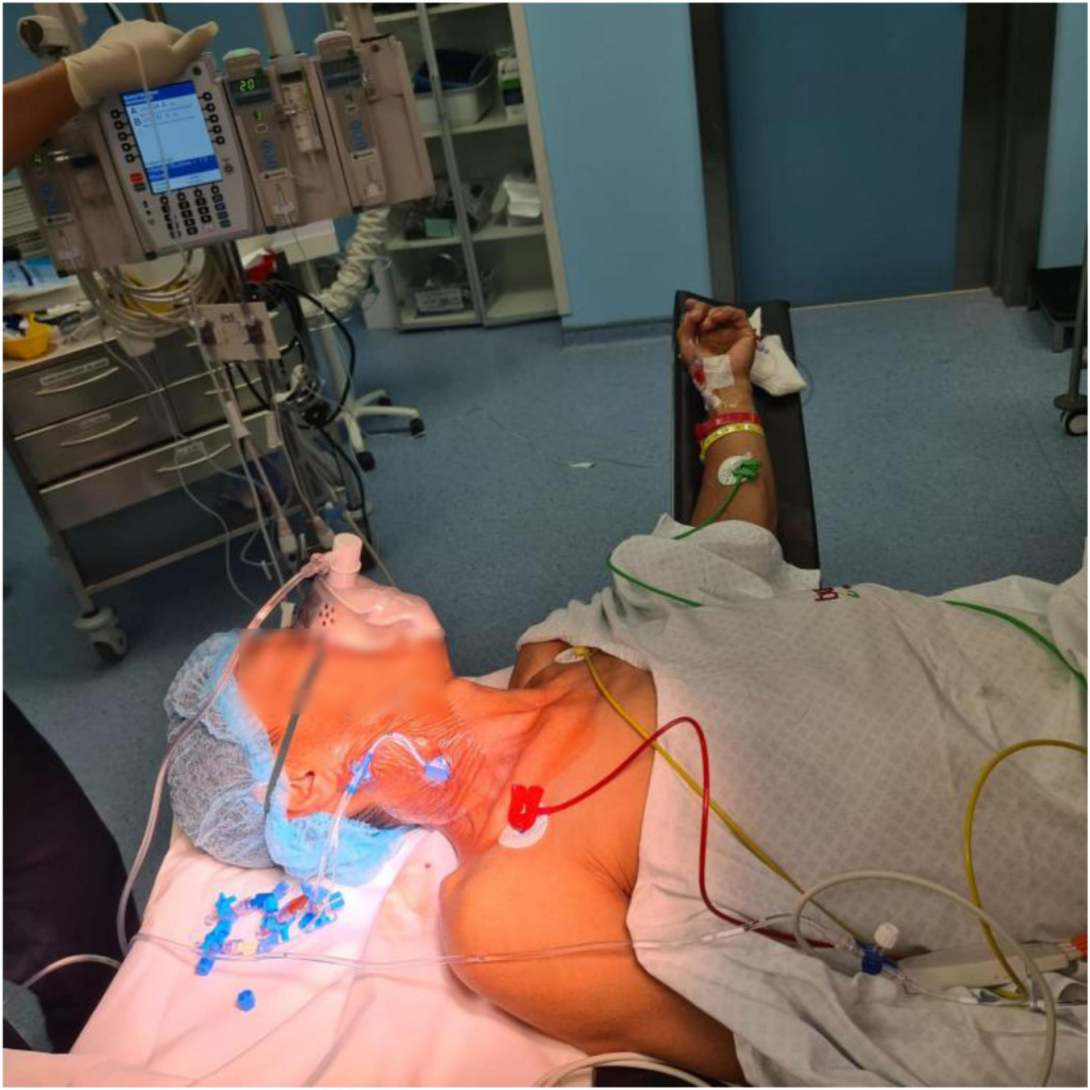
Patient after placement of thoracic epidural catheter receiving supplemental oxygen via a face mask, with a central venous catheter placed in the right internal jugular vein and an arterial line in the left radial artery for continuous hemodynamic monitoring.

Patients underwent continuous monitoring utilizing standard equipment, which included blood pressure measurement, pulse oximetry, electrocardiography, temperature assessment, and end-tidal CO_2_ analysis. Before the procedure, a central line catheter was inserted in the internal jugular vein and patients were administered a fluid load of 1 L of Ringer's lactate, in addition to 1 mg of midazolam and 50 µg of fentanyl. The epidural catheter was inserted under strict sterile conditions. After positioning the patient in the sitting position, the insertion site (T6–T7 interspace) was disinfected with chlorhexidine solution and sterile fenestrated draping was applied. Using an 18G Tuohy needle, the epidural space was identified via the **loss-of-resistance** technique. A **test dose of 3 ml of 2% lidocaine** was administered to exclude intrathecal or intravascular placement. A sterile catheter was then advanced **4 cm** into the epidural space. The catheter was then secured with sterile dressings. Then, an 8 ml bolus of 0.5% Ropivacaine was administered, succeeded by 150 µg morphine in the epidural space. A continuous infusion of 0.25% Ropivacaine was subsequently maintained at a rate of 7 ml per hour. An arterial line was inserted into the left radial artery, and to maintain a mean arterial pressure above 65 mmHg, a continuous infusion of noradrenaline at an average dosage of 0.12 ± 0.06 mcg/kg/min was used to control sympathetic blockade-induced hypotension. Sedation involved low-dose midazolam or dexmedetomidine as needed (Figure 1). After surgery, all patients received 4 mg intravenous ondansetron to prevent postoperative nausea and vomiting (PONV).

#### 2.1.2 General anesthesia protocol with endotracheal intubation and mechanical ventilation

Patients were continuously monitored using standard equipment, including blood pressure measurement, pulse oximetry, electrocardiography, temperature assessment, and end-tidal CO₂ analysis. Prior to induction, intravenous access was secured and patients received a fluid load of 1 L of Ringer's lactate, along with appropriate premedication (2 mg of midazolam and 50 µg of fentanyl). Following preoxygenation, anesthesia was induced using intravenous agents—typically propofol—and neuromuscular blockade was achieved with a suitable agent (rocuronium) to facilitate endotracheal intubation. Endotracheal intubation was then performed under direct visualization of both vocal cords, and anesthesia was maintained with a volatile agent (sevoflurane) in an oxygen–air mixture, supplemented with additional opioids as needed. Standard monitoring was continuously maintained throughout the procedure. In this group, an epidural catheter was also inserted prior to intubation using the same technique as mentioned above and maintained in both groups for 3–5 days to provide postoperative analgesia, and the patients were extubated and then moved to the intensive care unit for short-term observation. The postoperative pain management was accomplished using a PCEA with ropivacaine 0.2% and fentanyl (2 mcg/ml), basal rate 6–8 ml/hr, 3 ml bolus, and 15 min lockout. This is the institutional standard for pancreatic surgeries under ERAS protocol.

### 2.2 Surgical techniques of pylorus-preserving pancreaticoduodectomy (PPPD)

**Figure 2:**
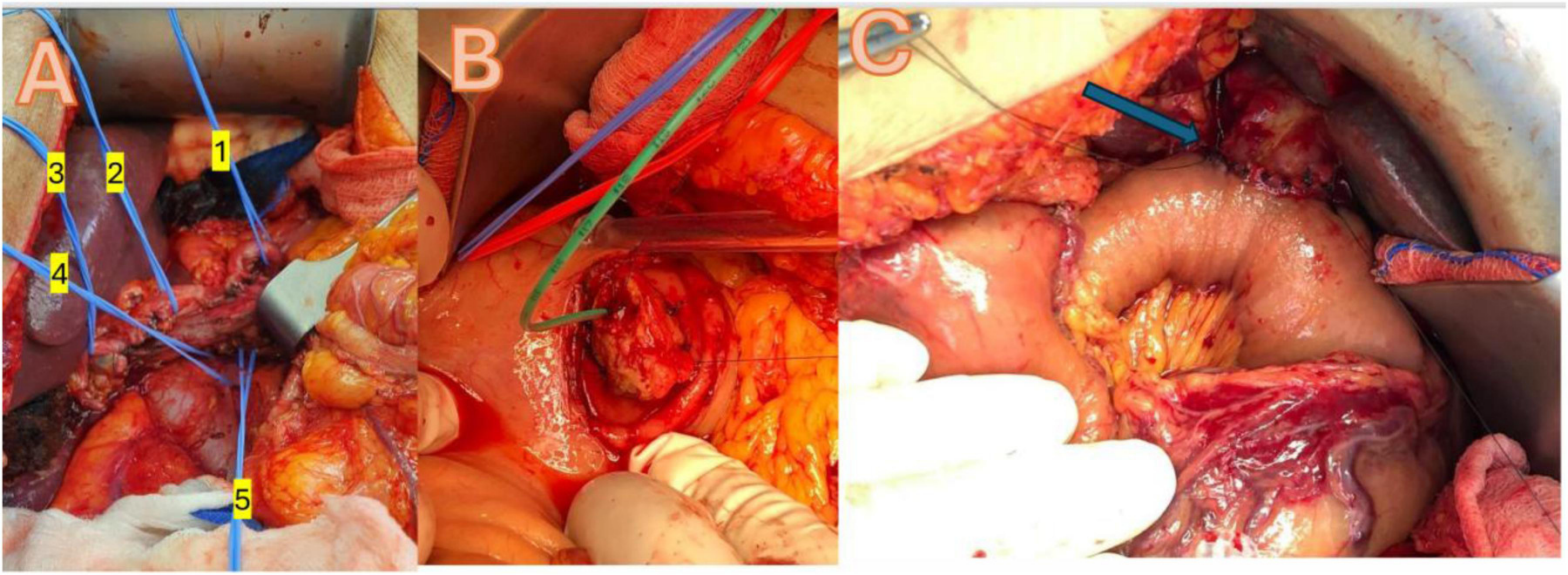
**(A)**, Extensive dissection with looping of all important vascular structures: 1 = splenic artery, 2 = common hepatic artery, 3 = proper hepatic artery, 4 = left renal vein, 5 = mesenteric superior artery; **(B)**, pancreatic tail inavaginated through posterior wall of stomach with green pancreatic duct tube; **(C)**, blue arrow shows completed roux and Y hepaticojejunostomy with PDS 4.0 in interrupted single stitch technique.

A median laparotomy or roof-top incision was used to gain access, with a Condor Abdominal Retractor employed to optimize exposure. Dissection was facilitated by the use of Harmonic and LigaSure energy devices, ensuring precise tissue division and hemostasis. Vascular clips were applied for vessel control, and anastomoses were performed using PDS 3.0 and 4.0 sutures. Reconstruction is achieved through a pancreatico-gastrostomy, incorporating a duct tube to maintain pancreatic duct patency and promote effective drainage, all while preserving the pylorus for optimal gastrointestinal continuity (Figure 2).

### 2.3 Data collection

Data collected included demographic variables (age, gender, ASA classification), operative details (procedure type, duration, conversion rates), postoperative outcomes (hospital stay, complications, ICU admissions), and oncologic parameters (R0 resection status, lymph node yield, histological tumor stage). Postoperative complications were graded using standard criteria, and pain management efficacy was assessed via patient-reported pain scores and analgesic requirements.

### 2.4 Statistical analysis

Student's *t*-test was used to compare the mean values of continuous variables. When the sample size was small, we used the chi-squared test to compare continuous variables, and Fisher's exact test for categorical variables. Non-parametric variables were distinguished between groups using the Mann–Whitney test. The statistical test ANOVA was used to assess whether there were any statistically significant differences among the mean values of the three groups. SPSS 29.0 was used for all statistical testing (IBM, SPSS® Chicago, IL, USA). To draw conclusions from the data, a *p*-value of less than 0.05 was considered statistically significant.

## 3 Results

### 3.1 Patient demographics and operative data

A total of 22 patients (15 males and 7 females) with a mean age of 50.7 years (range 27–74) were included. No significant differences in preoperative demographics, including ASA classification, were observed between the GA and EA groups, even though patients in the GA group had a higher body mass index (Table 1). The immunological parameter showed a trend towards the EA group without reaching statistical significance (Table 2).

**Table 1:** Comparison of age and preoperative body mass index (BMI) of the GA and EA groups.

Patient characteristics	General anesthesia	Epidural anesthesia	Total
Gender female/male (*n*)	6/8	1/7	7/15
Age in years	46.9	57.4	
Average preoperative BMI	28.1^a^ (*p* = 0.001)	22.7	
Average ASA classification	3	3	

a Denotes a statistically significant difference between the groups, using the t-test for means comparison test. (*n*) is the absolute number of cases.

**Table 2:** Comparison of immunological parameter of the GA and EA groups on first postoperative day.

Immunological parameters	Type of anesthesia	*N*	Mean	Std. deviation	*P*-value
Procalcitonin	General anasthesia	14	3.0464	3.39459	0.055
	Epidural anesthesia	5	20.05	32.01434	
C-reactive protein (CRP)	General anasthesia	14	129.584	59.6211	0.388
	Epidural anesthesia	8	162.964	97.497	
White blood cell count (WBC)	General anasthesia	14	16.4914	4.25454	0.899
	Epidural anesthesia	8	16.2188	5.64995	

Group statistics using independent t-test.

**Figure 3:**
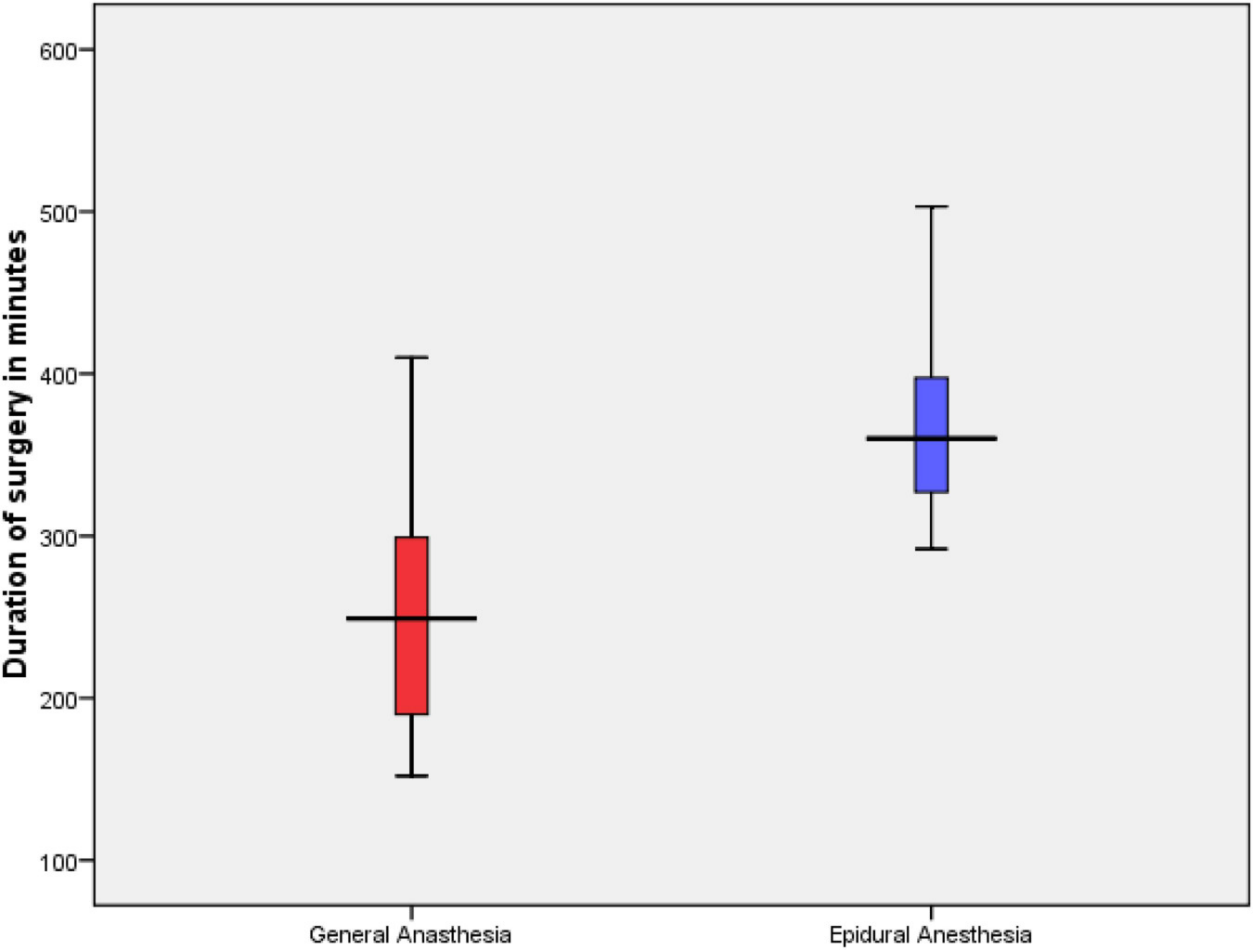
Comparison of duration of surgery of the GA and EA groups. Group statistics using independent t-test, *p* = 0.022.

In the GA group, 13 patients underwent PPPD and 1 underwent total pancreatectomy with splenectomy (TPS). In the EA group, six patients underwent PPPD and two underwent TPS. The average surgery time was significantly longer in the EA group (Figure 3). However, the average hospital stay was 17.6 days (range 8–44) without statistical difference between the two groups; moreover, the specific biochemical parameter did not differ between the two groups (Table 3).

**Table 3:** Comparison of early clinical outcome parameter of the GA and EA groups during hospital stay. Group statistics using independent t-test.

Clinical & biochemical parameters	Type of anesthesia	*N*	Mean	Std. deviation	*P*-value
	Epidural anesthesia	8	20.7	11.1	
Length of stay in days	General anasthesia	14	15.93	9.903	0.302
	Epidural anesthesia	8	20.75	11.132	
Serum amylase POD 1	General anasthesia	14	147.07	195.173	0.702
	Epidural anesthesia	8	182.25	220.278	
BILIRUBIN POST OP	General anasthesia	13	12.892	13.037	0.066
	Epidural anesthesia	8	32.838	33.3763	
Drain amylase POD 1	General anasthesia	11	1,042.27	1,566.076	0.306
	Epidural anesthesia	6	343.17	266.943	

### 3.2 Feasibility and safety outcomes

In the EA group, seven patients completed the procedure entirely under epidural anesthesia without conversion (feasibility outcome), while one patient undergoing total pancreatectomy with splenectomy required conversion to general anesthesia due to intraoperative respiratory acidosis. The safety outcome included no mortality or major cardiopulmonary complications being observed during hospitalization, aside from one patient in the GA group who developed COVID-19 and required prolonged ventilation for 10 days before recovery. Overall, surgical complications were minimal: two GA patients developed pancreatic fistula type B, one EA patient experienced a biliary leak (all managed conservatively), and the COVID-positive GA patient underwent revision laparoscopy on postoperative day 30 for an iatrogenic bowel perforation following interventional radiology drain insertion for chylous ascites.

### 3.3 Oncological outcomes and postoperative complications

Therefore, short-term surrogate oncologic indicators such as R0 resection rate, number of harvested lymph nodes, lymph node ratio, and perioperative tumor marker trends were deliberately included in the analysis. These parameters reflect oncologic adequacy and early treatment response, allowing us to evaluate whether epidural anesthesia supports, or at the very least does not compromise, oncologic quality in patients undergoing major pancreatic head resections. All patients achieved an R0 resection. Moreover, the EA group demonstrated a significantly higher lymph node yield and a greater number of lymph node metastases (Table 4).

**Table 4:** Comparison of early oncological outcome parameter of the GA and EA groups during hospital stay. Group statistics using independent *t*-test.

Oncological parameters	Type of anesthesia	*N*	Mean	Std. deviation	*P*-value
Preoperative CA 19-9 in U/ml	General anasthesia	13	83.74	130.47	0.07
	Epidural anesthesia	8	256.13	281.33	
Postoperative CA 19-9 in U/ml	General anasthesia	12	38.52	62.61	n.s
	Epidural anesthesia	7	71.58	84.84	
Preoperative CEA in ng/ml	General anasthesia	13	2.98	2.70	n.s
	Epidural anesthesia	8	5.37	8.70	
Postoperative CEA in ng/ml	General anasthesia	13	2.42	1.48	n.s
	Epidural anesthesia	5	3.46	4.58	
Size of the tumor in cm	General anasthesia	14	4.13	2.63	n.s
	Epidural anesthesia	8	3.64	1.70	
Number of lymph node metastases	General anasthesia	14	1.14	2.21	0.005
	Epidural anesthesia	8	5.63	4.50	
Number of lymph node yields	General anasthesia	14	11.50	10.06	0.022
	Epidural anesthesia	8	27.63	20.65	

## 4 Discussion

Pancreatic cancer remains one of the most lethal malignancies, with recurrence and metastasis posing significant challenges even after surgery. Emerging evidence indicates that the anesthetic techniques employed during cancer surgery may influence oncological outcomes by modulating the body's immune response, stress levels, and inflammatory processes, potentially affecting the risk of cancer recurrence and metastasis (19).

Our study demonstrates that pyloric-preserving pancreaticoduodenectomy performed under epidural anesthesia without endotracheal intubation is both feasible and safe in selected pancreatic cancer patients. The high rate of successful EA completion—with only one conversion due to respiratory acidosis—and even better oncological outcomes (as measured by tumor marker drop postoperatively, rate of lymph node metastasis, and total number of lymph node yields) indicate that EA does not compromise the surgical radicality required for effective treatment.

A major advantage of EA is its capacity to mitigate the perioperative neuroendocrine stress response. GA with intubation elevates catecholamines and inflammatory cytokines, which may impair natural killer cell function and promote tumor dissemination. Additionally, GA-induced metabolic alterations, as demonstrated by recent metabolomic studies, may further influence tumor behavior. In contrast, EA reduces these deleterious responses, thereby establishing a more favorable immunologic and metabolic environment. Recent evidence supports the beneficial role of EA in optimizing perioperative outcomes after pancreatic surgery (20).

However, Hou-Choun et al. reported that propofol anesthesia was associated with improved survival in open pancreatic cancer surgery compared to desflurane anesthesia, although their study was based on a limited sample size (21). Ren et al. found no significant difference in overall survival and disease-free survival between total intravenous anesthesia and volatile anesthesia (22). Furthermore, long-term outcomes from the PAKMAN randomized study revealed no significant survival difference between patients receiving perioperative thoracic epidural analgesia and those managed with patient-controlled intravenous analgesia (23).

Our study showed that patients in the EA group had a tendency towards a longer hospital stay compared to those in the GA group, although this difference was not statistically significant, and there was no associated increase in morbidity. These findings align with previous research, which reported that epidural analgesia was linked to a prolonged length of stay—most notably affecting early discharge in patients undergoing open pancreaticoduodenectomy and distal pancreatectomy (24).

Our study demonstrated that EA is a safe and feasible technique for complex pancreatic head resections. No major technical drawbacks were observed, and EA was successfully performed in all cases except one, which required conversion to GA at the end of surgery due to respiratory acidosis. Our cohort, consisting solely of patients with malignant pancreatic head cancer, is comparable to many series reported in the literature. The mean operative time of 300 ± 87 min was within the range of previously published data and was not adversely affected by the absence of neuromuscular blockade (16, 25). Additionally, the use of EA did not increase the risk of bleeding or compromise hemodynamic stability. Despite minor challenges related to the patient's breathing, no significant issues occurred during the most complex surgical maneuvers, including the management of major vessels and lymphadenectomies, as shown in Figure 2.

Effective pain control was achieved in all cases, enabling early mobilization and timely resumption of oral intake. In both groups no patient died within the first 90 days after discharge, although long-term survival data were unavailable due to the multicultural composition of our cohort. There were no significant differences in postoperative inflammatory or tumor markers between the groups. Only one patient required conversion from EA to GA at the end of the procedure, likely due to an anesthesiologist handover driven by hospital working hours policy rather than respiratory acidosis, and this conversion was performed smoothly. The overall cohort experienced a longer hospital stay compared to more recent studies (26–27), primarily due to the pivotal nature of this study and the wide geographical referral of patients, which necessitated a more cautious approach to discharge. However, the length of stay remained comparable to that reported in studies from the past decade (28).

Our study is the first to compare anesthesia type with lymph node yield and metastatic ratio in major abdominal procedures such as pancreatic head resection. While most research has focused on surgical technique, specimen processing, or neoadjuvant therapy as determinants of lymph node yield, our findings demonstrate that even major pancreatic resections yield more lymph nodes and higher lymph node metastasis detection when performed under epidural anesthesia—without endotracheal intubation or muscle relaxants—indicating that this anesthesia approach does not compromise oncological radicality. In selected cases with tumor involvement of both the pancreatic head and body, total pancreatosplenectomy (TPS) was performed based on intraoperative frozen section analysis of resection margins. Given that TPS was more frequently performed in the EA group, this may have contributed to differences in total lymph node yield between groups and lower rate of postoperative pancreatic fistula.

A recent study by Leoni et al. (29) advocated for the use of neuraxial awake anesthesia in 16 emergency laparotomies for acute intestinal disorders during the COVID-19 pandemic to minimize SARS-CoV-2 aerosolization associated with general anesthesia. All patients in the study underwent resections of the small bowel or left colon, typically executed through a periumbilical or lower midline incision, thereby avoiding the need for upper abdominal laparotomy, which may compromise ventilation and impact the subcostal nerves, which is the case in our series. Furthermore, the damage-control procedures exhibit notable differences compared to complex pancreatic surgeries, which require extensive retroperitoneal dissection around major vascular structures such as the superior mesenteric artery, portal vein, and celiac axis, as well as intricate pancreaticobiliary and intestinal reconstruction. Leoni et al. concentrated on life-saving outcomes; however, their study did not consider oncological issues, and it remains unspecified whether some emergency cases involved malignancy. Conversely, this study focuses on oncological outcomes.

Finally, a propensity-weighted analysis in renal cell carcinoma by Yen et al. illustrates that the association between anesthetic modality and oncological outcomes may vary by tumor type. These diverse findings underscore the complex interplay between anesthetic technique and oncological outcomes (30).

This study is limited by its small sample size, retrospective design, and potential selection bias. Patients in the epidural anesthesia (EA) group had significantly lower BMI compared to the general anesthesia (GA) group, which could influence perioperative outcomes. Although EA may have immunomodulatory benefits compared to GA, our small retrospective study was not designed to assess inflammatory or immune responses, so no conclusions on this aspect can be drawn. Consequently, our findings should be interpreted as exploratory and focused primarily on feasibility and safety rather than efficacy.

## 5 Conclusions

Our study supports the safety and feasibility of performing major pancreatic head resections for cancer under epidural anesthesia without endotracheal intubation. We found that this approach does not significantly increase morbidity or mortality compared to procedures conducted under general anesthesia. Additionally, short-term oncological outcomes, including complete tumor resection and the percentage reduction in tumor markers postoperatively, were similar between the two groups. The EA group showed a higher incidence of lymph node metastases and a greater total lymph node yield during major pancreatic resection, indicating potentially improved short-term oncological outcomes in this cohort.

However, to establish definitive conclusions regarding long-term outcomes, larger randomized controlled trials are needed. These studies will provide further insights into the efficacy and long-term oncological benefits of epidural anesthesia in pancreatic surgery, ultimately guiding clinical practice and optimizing patient outcomes.

## Data Availability

The original contributions presented in the study are included in the article/Supplementary Material, further inquiries can be directed to the corresponding author.
